# Clinical course and management of 73 hospitalized moderate patients with COVID-19 outside Wuhan

**DOI:** 10.1371/journal.pone.0249655

**Published:** 2021-05-13

**Authors:** Xiaojuan Peng, Qi Liu, Zhaolin Chen, Guiyan Wen, Qing Li, Yanfang Chen, Jie Xiong, Xinzhou Meng, Yuanjin Ding, Ying Shi, Shaohui Tang

**Affiliations:** 1 Department of Endocrinology, Affiliated Hospital (Clinical College) of Xiangnan University, Chenzhou, Hunan, P. R. China; 2 Department of Infectious Diseases, The First People’s Hospital of Xiaochang County, Hubei, P. R. China; 3 Department of Gastroenterology, The First Affiliated Hospital, Jinan University, Guangzhou, Guangdong, P. R. China; 4 Department of Interventional vascular surgery, Affiliated Hospital (Clinical College) of Xiangnan University, Chenzhou, Hunan, P. R. China; 5 Department of Cardiology, The First People’s Hospital of Xiaochang County, Hubei, P. R. China; 6 Department of Hepatobiliary surgery, The First People’s Hospital of Xiaochang County, Hubei, P. R. China; University Magna Graecia of Catanzaro, ITALY

## Abstract

Moderate cases account for the majority in hospitalized patients with severe acute respiratory syndrome coronavirus 2 (SARS-CoV-2) infection and can also progress to severe/critical condition. Here, we investigated the clinical course and management of hospitalized moderate SARS-CoV-2 patients. The medical records and follow-up data were analyzed from the SARS-CoV-2 patients outside Wuhan. A total of 73 moderate patients (38 men, 35 women) were included, with median age of 47.0 (38.5–57.5) years. Among them, only one patient (1.4%) died using active treatment to improve symptoms. The median duration of the four main symptoms cough, fever, chest tightness, and fatigue were 11.0, 8.0, 11.0, and 7.0 days, respectively; the median duration of the positive nucleic acid test (NAT) results for SARS-CoV-2 was 16.5 days; the median hospitalization time was 25.0 days in 72 moderate survivors. The duration of cough and fever was positively correlated with the duration of the positive NAT results. On admission, 50% had lymphopenia; less than 30% had abnormal blood biochemistry findings involving hyperglycemia, liver function and myocardial enzymes. At discharge, the laboratory indexes were substantially improved. Two weeks after discharge, 5.6% survivors experienced a recurrence of the positive NAT results. Moderate SARS-CoV-2 patients have a good prognosis by the active treatment. A small proportion of the recovered moderate patients still may be virus carriers and require an additional round of viral detection.

## Background

The outbreak of novel coronavirus diseases (COVID-19), which is caused by severe acute respiratory syndrome coronavirus 2 (SARS-CoV-2) infection, has been spreading rapidly worldwide and become a global pandemic [1]. The clinical spectrum of COVID-19 ranges from mild to critically ill cases. In addition, asymptomatic cases with SARS-CoV-2 infection have been reported, which are diagnosed based on positive viral nucleic acid test results, but without COVID-19 symptoms and significant abnormalities on chest imaging [2]. The prevalence of asymptomatic carriers varies from 4.0% to 21.3% (2–4) in cases infected with SARS-CoV-2 [3–5]. Most patients with COVID-19 are nonsevere, and severe patients can progress rapidly to critical condition, including acute respiratory distress syndrome (ARDS), multi-organ dysfunction syndrome (MODS) and even death [6–8]. COVID-19 has contributed to an enormous adverse impact globally.

According to the diagnostic and treatment guideline for COVID-19 issued by Chinese National Health Committee (version 3–6) (http://www.nhc.gov.cn/), the clinical classification of COVID-19 severity includes four clinical types: mild, moderate, severe, and critical, of which moderate cases account for the majority in hospitalized patients in China [9, 10]. Moderate patients with COVID-19 can also progress to severe/critical condition [11, 12]. It is obvious that inhibiting COVID-19 progression can significantly improve the prognosis of the disease. Previous reports have described the general epidemiological findings and clinical characteristics of patients with COVID-19, and differences between subjects with the mild and severe diseases [6, 7, 13, 14]. In the present study, we focused on the clinical course and management of hospitalized moderate patients with COVID-19 in a non-Wuhan area (Xiaochang County) of Hubei Province in order to provide information for understanding the development, progression, and prognosis of the disease.

## Methods

### Ethical approval

The study was approved by the medical ethics committee of the First People’s Hospital of Xiaochang County, Hubei Province, China (No. 2020–05).

Besides statistical data including means, medians, P values and patient numbers, we also publicly provided individual clinical data in our figures (e.g. Fig 2). Nevertheless, there are some data from patient participants which refer to sensitive data we could not provide according to the research ethics and applicable local laws. However, we still would like to share the data as many as possible. Therefore, if there are some audiences who are interested in our research and would like to get more data, please contact to corresponding authors freely. We will consult with an ethics committee to ensure data are shared in accordance with participant consent and all applicable local laws.

### Patients

We retrospectively analyzed the medical records and follow-up data during hospitalization and after discharge from the patients with COVID-19 consecutively admitted to the Department of infection, the First People’s Hospital of Xiaochang County, Xiaogan City, Hubei Province from January to May, 2020. The hospital, which is about 90 km away from Wuhan, China, is the designated hospital for the hospitalization of patients with COVID-19. The following criteria were required to meet for hospital discharge (http://www.nhc.gov.cn/): (1) normal temperature for at least 3 days, (2) significant improvement in respiratory symptoms, (3) obvious signs of absorption of acute exudative lesions on chest computed tomography (CT) images, and (4) 2 consecutive negative nucleic acid test results for SARS-CoV-2 with at least one-day interval. After the recovered COVID-19 patients were discharged, they were transferred to designated quarantine places for a 14-day medical observation, and then underwent another 14-day self-imposed quarantine at home. During follow-up, the patients were asked to conduct the nucleic acid test (NAT) for SARS-CoV-2 14 days after discharge and chest CT scans 28 days after discharge.

### Data collection

Epidemiological, demographic, clinical, laboratory, and medical imaging findings and management information from patients’ medical records and follow-up data were collected. SARS-CoV-2 pneumonia was diagnosed based on clinical symptoms with typical changes in chest CT image and positive for the nucleic acids of SARS-CoV-2. The clinical classification of COVID-19 severity includes four types (http://www.nhc.gov.cn/): mild, moderate, severe, and critical. Mild cases refer to having only mild symptoms and no manifestation of pneumonia in imaging. Moderate cases refer to having fever, respiratory tract symptoms, and manifestation of pneumonia in imaging. Severe cases meet any of the following signs: a) respiratory distress, respiratory rate≥30 beats/ min; b) in the resting state, finger oxygen saturation ≤93%; c) arterial blood oxygen partial pressure (PaO_2_) / oxygen concentration (FiO_2_) ≤ 300mmHg (1mmHg = 0.133kPa). Critical cases meet one of the following criteria: (a) respiratory failure with mechanical ventilation; (b) shock; (c) combination with other organ failure, with ICU monitoring and treatment. The nucleic acid testing for SARS-CoV-2 was performed using quantitative RT-PCR on samples from the respiratory tract (oropharyngeal swab samples) by the Xiaogan Center for Disease Control and Prevention, which is the designated laboratory for SARS-CoV-2 test.

### Statistical analysis

Categorical data were expressed as number (%) and evaluated by χ2 or Fisher’s exact test; continuous data were expressed as median (interquartile range (IQR)) and evaluated by Mann-Whitney *U* test; linear correlation was evaluated by Pearson correlation analysis. A two-sided α of less than 0·05 was considered statistically significant. All the statistical analyses were performed with SPSS (version 26.0).

## Results

### Demographics and clinical characteristics

**Fig 1:**
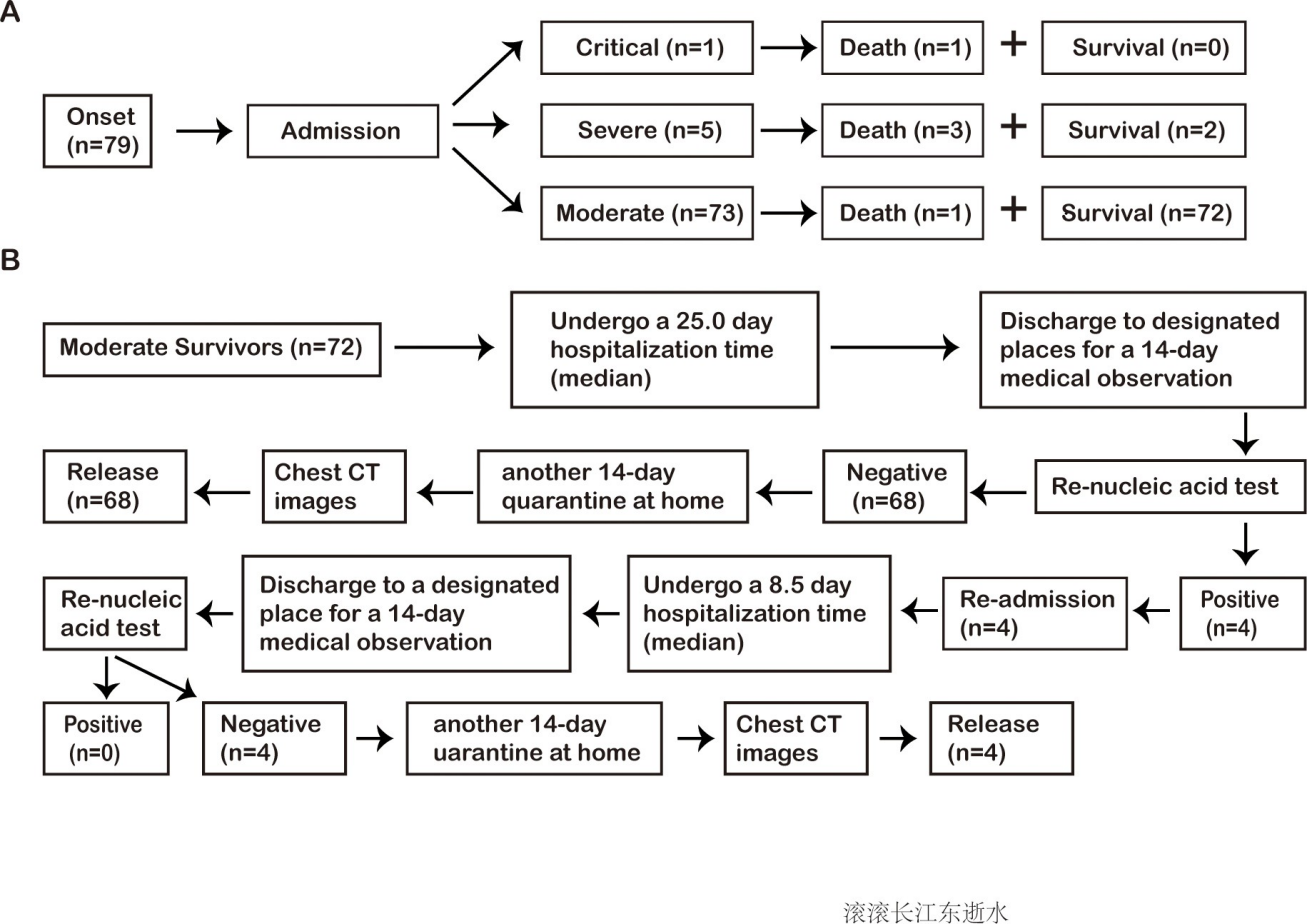
The prognosis of 79 patients with COVID-19 including 73 moderate cases, 5 severe cases, 1 critical case (A), and the flow chart of the clinical management for the 72 moderate survivors with COVID-19 (B). COVID-19, novel coronavirus diseases.

A total of 79 patients with COVID-19 were included in this study (42 men, 37 women), with median age of 48.0 (39.0–59.0) years. Among them, there were 73 moderate cases (38 men, 35 women) with median age of 47.0 (38.5–57.5) years, 5 severe cases, 1 critical case, and no mild case on admission. The mortality rate (66.7%, 4/6) in severe/critical patients was higher than that (1.4%, 1/73) in moderate patients, whereas there were no significant differences with respect to exposure history, occupation, smokers, comorbidity and so on between moderate patients and severe/critical patients (S1 Table, Fig 1A).

The most common symptoms at disease onset were cough (72.6%), fever (38.4%), chest tightness (34.2%), fatigue (21.9%), and gastrointestinal symptoms (19.2%), and other less common symptoms included dizzy/ headache (12.3%), nasal obstruction (8.2%), chill (2.7%), and runny nose (1.4%) in 73 moderate patients. Only one case developed complications (acute respiratory distress syndrome and acute heart failure) in 73 moderate patients that was less common compared with 6 severe/critical patients (S2 Table).

**Fig 2:**
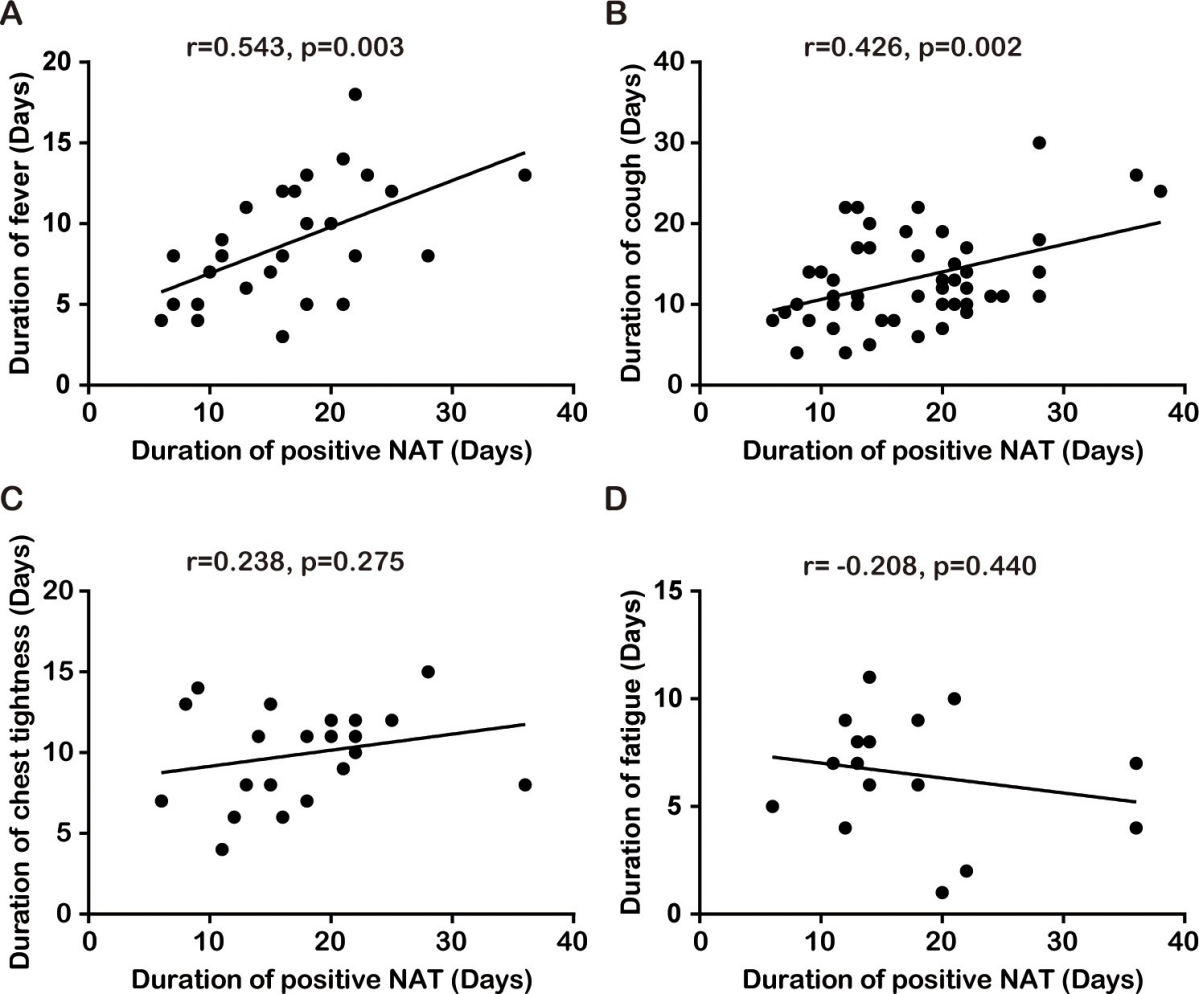
Linear correlation analysis between the duration of positive NAT results and the duration of cough (A), fever (B), chest tightness (C), and fatigue (D). NAT results, nucleic acid test results for SARS-CoV-2.

The main symptoms cough, fever, chest tightness, and fatigue during hospitalization lasted for 11.0 (9.3–17.0), 8.0 (5.0–12.0), 11.0 (8.0–12.0), and 7.0 (4.5–8.5) days, respectively, and the duration of positive nucleic acid test (NAT) results for SARS-CoV-2 was 16.5 (12.0–22.0) days in 72 moderate survivors with COVID-19. The median length of hospital stay was 25.0 (18.0–29.5) days; the median time from disease onset to discharge was 29.5 (25.0–36.5) days (Table 1). The duration of cough (r = 0.426, P = 0.002) and fever (r = 0.543, P = 0.003) was positively correlated with the duration of the positive NAT results, but the duration of chest tightness (r = 0.238, P = 0.275) and fatigue (r = -0.208, P = 0.440) was not correlated with the duration of the positive NAT results in 72 moderate patients (Fig 2).

**Table 1:** Clinical course and follow-up outcome of 72 moderate survivors with COVID-19.

Items	Values
Duration of main symptoms during hospitalization	
Cough-days	11.0(9.3–17.0)
Fever-days	8.0(5.0–12.0)
Chest tightness-days	11.0(8.0–12.0)
Fatigue-days	7.0 (4.5–8.5)
Dizzy/Headache-days	5.0 (2.5–7.5)
Nasal obstruction-days	5.5 (3.5–8.3)
Gastrointestinal symptoms-days	4.0 (3.0–6.0)
Duration of positive NAT results-days	16.5 (12.0–22.0)
Re-positive NAT results 2 weeks after discharge-n (%)	4 (5.6)
Chest CT findings on admission	
Bilateral viral pneumonia-n (%)	52 (72.2)
Unilateral viral pneumonia-n (%)	20 (27.8)
Chest CT images on discharge	
Recovered completely-n (%)	15 (20.8)
Improved significantly-n (%)	57 (79.2)
Chest CT images 4 weeks after discharge (n = 68)	
Recovered completely-n (%)	49 (72.1)
Improved further-n (%)	19 (27.9)
Treatment regimen	
Antiviral treatment-n (%)	72 (100.0)
Corticosteroid treatment-n (%)	25 (34.7)
Antibacterial treatment-n (%)	32 (44.4)
Oxygen support by nasal cannula-n (%)	40 (55.6)
Traditional chinese medicine-n (%)	66 (91.7)
Length of hospital stay-days	25.0 (18.0–29.5)
Time from disease onset to discharge-days (n = 68)	29.5 (25.0–36.5)
Time from disease onset to discharge-days (n = 72)	29.5 (25.0–36.5)

Data are shown as n (%) or median (IQR). COVID-19, coronavirus disease 2019; NAT, nucleic acid test for SARS-CoV-2; IQR, interquartile range.

### Laboratory and imaging findings in 72 moderate survivors with COVID-19

On admission, most patients had the normal range of leucocytes (79.2% cases), neutrophils (86.1% cases), and neutrophil percentage (72.2% cases), but a small minority of patients had decreased leucocytes (19.4% cases) and increased neutrophil percentage (26.4% cases). Lymphocytes and lymphocyte percentage were below the normal range in 50.0% of the patients and 40.3% of the patients, respectively. Platelets were within the normal range in 88.9% of the patients, and a small number of patients had decreased platelets (11.1% cases) (Table 2).

**Table 2:** Laboratory characteristics of 73 moderate patients with COVID-19.

Items	Patients on admission (n = 73)	Patients on discharge (Except one death, n = 72)	P-value
**Blood routine**			
Leucocytes (×10^9^/L; normal range 3.5–9.8)	4.7 (3.8–5.7)	5.1 (4.2–6.4)	0.093
Increased-n (%)	1 (1.4)	1 (1.4)	1.000
Decreased-n (%)	14 (19.2)	5 (6.9)	0.027
Neutrophils (×109/L; normal range 1.8–6.3)	3.3 (2.4–4.1)	3.2 (2.6–4.0)	0.856
Increased-n (%)	3 (4.1)	3 (4.2)	1.000
Decreased-n (%)	7 (9.6)	3 (4.2)	0.190
Neutrophil percentage-(%) (normal range 40–75)	69.8 (61.3–75.3)	62.4.8 (56.8–67.7)	0.004
Increased-n (%)	19 (26.0)	7 (9.7)	0.009
Decreased-n (%)	1 (1.4)	1 (1.4)	1.000
Lymphocytes (×10^9^/L; normal range 1.1–3.2)	1.08 (0.8–1.3)	1.3 (1.1–1.6)	0.000
Decreased-n (%)	37 (50.7)	19 (26.4)	0.004
Lymphocyte percentage-(%) (normal range 20–50)	21.4 (17.0–31.6)	27.1 (21.2–31.4)	0.022
Decreased-n (%)	30 (41.1)	13 (18.1)	0.003
Platelets (×10^9^/L; normal range 125–350)	193.0 (144.0–245.0)	234.5 (194.8–261.0)	0.002
Decreased-n (%)	8 (11.0)	1 (1.4)	0.033
Haemoglobin (normal range 115–150 g/L)	131.0 (118.5–142.0)	128.0 (120.0–134.8)	0.189
Decreased-n (%)	11 (15.1)	6 (8.3)	0.197
**Infection biomarkers**			
C-reactive protein (mg/L; normal range 0.0–8.0)	15.3 (3.6–29.0)	1.4 (0.8–4.2)	0.000
Increased-n (%)	48 (65.8)	9 (12.5)	0.000
Procalcitonin (ng/mL; normal range 0.0–0.5)	0.05 (0.05–0.07)	0.05 (0.05–0.06)	0.219
Increased-n (%)	3 (4.0)	0 (0.0)	0.245
**Coagulation function**			
Prothrombin time (s; normal range 9.0–15.0)	11.5 (11.0–12.3)	11.5 (10.6–12.1)	0.347
Increased-n (%)	0.0 (0.0)	0.0 (0.0)	-
Activated partial thromboplastin time (s; normal range 22.0–45.0)	26.9 (25.1–29.5)	26.5 (24.7–27.9)	0.060
Increased-n (%)	0.0 (0.0)	0.0 (0.0)	-
D-dimer (μg/mL; normal range 0.0–0.5)	0.08 (0.04–0.22)	0.08 (0.00–0.22)	0.903
Increased-n (%)	9 (12.3)	8 (11.1)	0.796
**Blood biochemistry**			
Fasting blood glucose (mmol/L; normal range 3.9–6.1)	5.2 (4.8–5.8)	4.8 (4.4–5.2)	0.000
Increased	16 (21.9)	5 (6.9)	0.016
Albumin (g/L; normal range 35.0–52.0)	38.3 (34.8–39.9)	36.1 (34.0–38.6)	0.025
Decreased-n (%)	20 (27.4)	27 (37.5)	0.213
Alanine aminotransferase (U/L; normal range 9–50)	19.8 (13.0–32.8)	24.2 (14.6–41.4)	0.110
Increased-n (%)	8 (11.0)	10 (13.9)	0.614
Aspartate aminotransferase (U/L; normal range 15–40)	21.6 (16.3–29.1)	18.6 (14.3–25.2)	0.035
Increased-n (%)	10 (13.7)	6 (8.3)	0.289
γ-Glutamyl transferase (U/L; normal range 11–61)	22.5 (16.1–35.8)	26.9 (17.9–46.0)	0.129
Increased-n (%)	8 (11.0)	10 (13.9)	0.614
Total bilirubin (μmol/L; normal range 5.1–23.0)	7.8 (6.4–9.9)	8.0 (6.2–10.6)	0.688
Increased-n (%)	3 (4.1)	2 (2.8)	1.000
Creatine kinase (U/L; normal range 26–174)	52.5 (35.2–79.9)	40.9(28.3–59.3)	0.003
Increased-n (%)	1.0 (1.4)	0 (0.0)	1.000
Creatine kinase-MB(U/L; normal range 3–25)	8.9 (6.7–12.4)	6.8 (5.7–10.3)	0.003
Increased-n (%)	1(1.4)	0 (0.0)	1.000
Lactate dehydrogenase (U/L; normal range 109–245)	182.2 (150.6–226.7)	170.2 (147.1–193.6)	0.031
Increased-n (%)	14 (19.2)	3 (4.2)	0.004
Serum creatinine (μmol/L; normal range 32–106)	66.0 (53.3–73.7)	59.4 (51.0–71.7)	0.158
Increased-n (%)	0 (0.0)	0 (0.0)	-
Blood urea nitrogen (mmol/L; normal range 1.5–7.5)	2.9 (2.2–3.6)	3.0 (2.4–3.7)	0.210
Increased-n (%)	0 (0.0)	0 (0.0)	-

Data are shown as median (IQR) or n (%). P values comparing patients on admission (n = 73) and patients on discharge (n = 72) are from χ^2^ test, Fisher’s exact test, or Mann-Whitney U test. COVID-19, coronavirus disease 2019; IQR, interquartile range.

More than half of the patients had a increased level of C-reactive protein (CRP) (65.3% cases), with median CRP level 14.1 mg/L (3.5–29.3). Procalcitonin (PCT) level was within the normal range in 95.8% of the patients (Table 2).

On admission, all patients showed the normal prothrombin time and the normal activated partial thromboplastin time. The D-dimer level was within the normal range in 87.5% of the patients (Table 2).

The fasting blood glucose (FBG) level was within the normal range in 79.2% of the patients, with 20.8% increased FBG on admission. Most of the patients had the normal liver function, with 27.8% decreased albumin (ALB) level and 13.9% increased aspartate aminotransferase (AST) level. Almost all patients had the normal range of creatine kinase (CK) and isoenzyme of creatine kinase (CKMB) levels, and most of the patients had the normal range of lactate dehydrogenase (LDH), with 19.4% increased LDH level. All patients had the normal range of blood urea nitrogen (BUN) and creatinine levels (Table 2).

At discharge, the abnormal laboratory results of the 72 survivors were significantly improved, but there were still some patients who had the abnormal laboratory findings (6.9% decreased leucocytes, 9.7% increased neutrophil percentage, 26.4% decreased lymphocytes, 18.1% decreased lymphocyte percentage, 12.5% increased CRP level, 6.9% increased FBG level, 37.5% decreased ALB level, 8.3% increased AST level, and 4.2% increased LDH level) compared with those on admission (Table 2).

**Fig 3:**
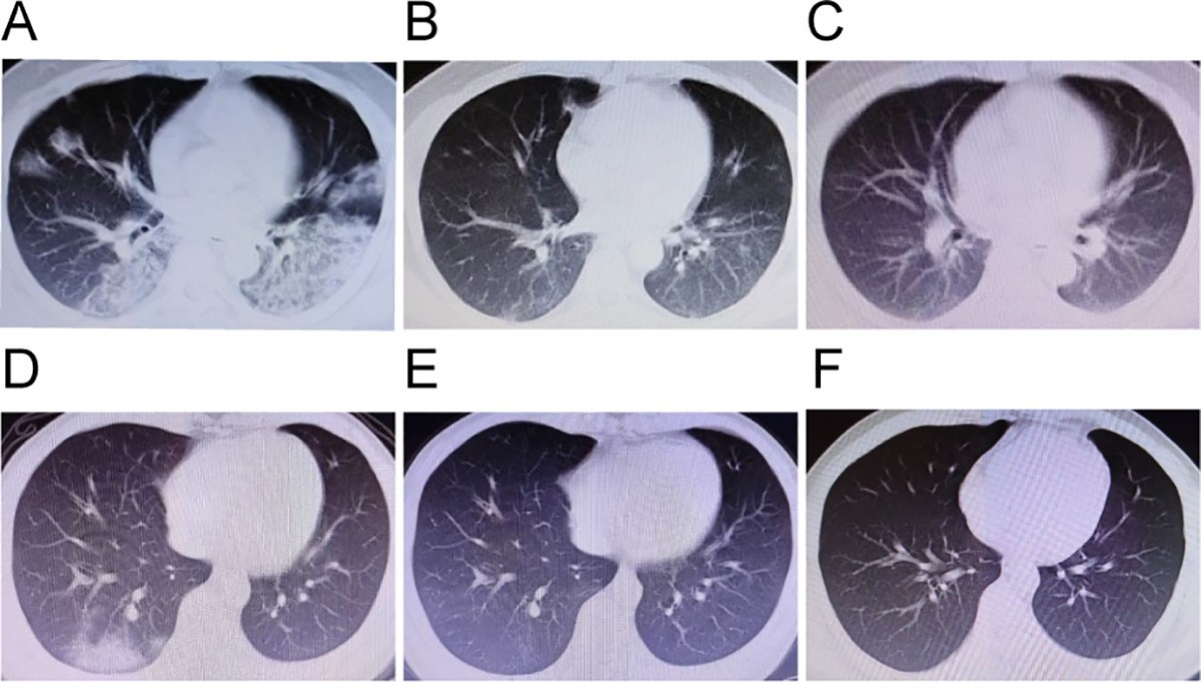
Chest CT images. The representative chest CT images of the moderate survivors. (A-C) A 38-year-old man showed bilateral ground-glass opacity co-existed with subsegmental areas of consolidation on admission (A), the above lesion improved significantly at discharge (B), and the above lesion improved further 4 weeks after discharge (C). (D-F) A 26-year-old man showed bilateral ground-glass opacity on admission (D), the above lesion improved significantly at discharge (E), and the above lesion absorbed completely 4 weeks after discharge (F).

On admission, 72.2% of the patients had bilateral viral pneumonia, and the remaining 27.8% patients had unilateral viral pneumonia in chest CT images. At discharge, the CT scans were negative in 20.8% of the patients, and the acute exudative lesions were improved significantly in the remaining 79.2% patients (Table 1). The representative chest CT images of the moderate survivors were presented in (Fig 3).

### Main treatment regimen

All moderate patients received antiviral therapy, including combination of interferon-α and lopinavir/ritonavir tablets or combination of interferon-α and abidol or combination of interferon-α and ribavirin; 34.7% of the moderate patients were given corticosteroid treatment (methylprednisolone or budesonide); 44.4% of the moderate patients were administered with empirical antibiotic treatment (cephalosporins or quinolones). 55.6% of the moderate patients received oxygen support by nasal cannula. Moreover, 91.7% of the moderate patients were administered with traditional Chinese medicine (Lianhua Qingwen granules or Qingfei Paidu decoction or Cold Dampness and Stagnant Lung decoction) (Table 1 and S3 Table).

### Follow up results in 72 moderate survivors with COVID-19

At the end of a 14-day medical observation, all survivors had no clinical symptoms, and the NAT showed that 4 (5.6%) of the patients had the re-positive results and 68 patients had the negative results after recovery in 72 moderate survivors. Chest CT scans were performed after undergoing another 14-day self-imposed quarantine at home in the 68 patients with the negative NAT results, and the result showed the acute exudative lesions were completely absorbed in 72.1% (49/68) of the patients, and the lesions were further improved in the remaining 27.9% (19/68) patients. At this point, the 68 patients were released from isolation (Table 1, Fig 1A).

The 4 patients with re-positive NAT results returned to the hospital for further treatment using the aforementioned regimen. They were 3 women and 1 man, with median age of 60.0 (41.3–72.8) years (S4 Table). All 4 patients had no clinical symptoms. On re-admission, almost all the laboratory results including blood routine, infection biomarkers, coagulation function, and blood biochemistry were within the normal range, and there were no significant changes on chest CT images compared with those at discharge (S5 Table). They underwent the median length of re-hospital stay of 8.5 (6.5–10.5) days, and were re-discharged after having 2 consecutive re-negative NAT results for SARS-CoV-2 with at least one-day interval. Subsequently, the 4 patients re-underwent a 14-day collective medical observation and another 14-day self-imposed quarantine at home. The 4 patients had the re-negative NAT results and the negative chest CT images 14 days and 28 days after re-discharge, respectively (Fig 1, S1 Fig).

## Discussion

In this study, we reported 79 patients with SARS-CoV-2 pneumonia, of who the clinical course and management of the 73 moderate cases were described for the first time. These patients came from Xiaochang County, Xiaogan City, a non-Wuhan area of Hubei Province, about 90 km away from Wuhan, who might be the second or third generation cases by human-to-human transmission of SARS-CoV-2 since the outbreak of COVID-19.

In our cohort of the 73 moderate patients, the most common symptoms included cough, fever, chest tightness, fatigue, and gastrointestinal symptoms (S2 Table), the occurrence rates of which were lower compared with the previous reports [9, 10, 14–18]. On admission, the main blood routine abnormality was lymphopenia in 50% of the moderate 72 survivors, perhaps suggesting that lymphocytes are the main target of SARS-CoV-2. More than half of the survivors had an increased CRP level, and less than 30% of the survivors had abnormal blood biochemistry findings involving hyperglycemia, liver function, and myocardial enzymes, rather than abnormal renal function and coagulation function in the 72 survivors (Table 2). Many studies have showed that lymphopenia is common in patients with COVID-19 [6, 7, 13, 15–17]; increased CRP levels are often reported in recent studies about COVID-19 [7, 15, 17]; several studies have reported hyperglycemia in patients with SARS-CoV-2 infection [7, 19]; decreased ALB, increased AST, and increased LDH levels are also found in patients with COVID-19 [6, 7, 13, 20]. The occurrence rates of these abnormal laboratory findings were higher compared with our corresponding results.

COVID-19 is a new infectious disease, and there were no effective drugs against SARS-CoV-2 at the time of this study. Therefore, in our cohort of the 73 moderate patients, treatment was focused on symptomatic improvement and preventing or inhibiting the disease progression, including nonspecific antiviral drugs, corticosteroid, empirical antibiotic drugs, oxygen support, and traditional Chinese medicine. Among the aforementioned treatments, corticosteroid may inhibit a cytokine storm and promote the absorption of exudative lesions, whereas antibiotic drugs could prevent or treat secondary bacterial infection [6, 7, 15].

In the 73 moderate patients, 72 cases recovered and 1 case died using the aforementioned treatments, and the mortality (1.4%) was lower than that of the first generation cases reported by Huang et al in Wuhan, who showed that 4% died in non-ICU patients with SARS-CoV-2 pneumonia [6]. It is possible that these treatments prevent or inhibit COVID-19 progression, or COVID-19 severity or population in the present study is different from that of cases reported by Huang et al. Of course, the small sizes of both studies make comparisons difficult. The median duration of the four main symptoms cough, fever, chest tightness, and fatigue were about 1–2 weeks, and the median duration of the positive NAT results was slightly more than 2 weeks during the hospitalization in 72 moderate survivors (Table 1). The median hospitalization time was almost 4 weeks, and the median time from onset of disease to discharge was about 1 month in 72 moderate survivors (Table 1). Linear correlation analysis showed there was a positive correlation between the duration of positive NAT results and the duration of cough or fever, rather than chest tightness and fatigue. These results suggest that the onset of the main symptoms such as cough and fever is due to SARS-CoV-2 replication, and these symptoms begin to resolve before the complete viral shedding. Kim et al reported that the median time of defervescence was 9 days (range, 3–18) after symptom onset in 28 patients with COVID-19 [11]. Wang et al showed that fever lasted for about 10 days and the positive NAT results for about 13 days in 88 surviving patients with COVID-19 [21]. These results are in part similar to our report.

At discharge, lymphocytopenia was significantly improved, with still about a quarter cases decreased lymphocytes, and the other abnormal blood routine parameters were also improved significantly, with less than 10% abnormal cases. Infection biomarker the CRP level decreased significantly, with still slightly more than 10% increased CRP level. The abnormal blood biochemistry results including FBG, liver function, and myocardial enzymes were improved significantly, with less than 10% abnormal cases. However, the ALB level was not improved, which seemed to further decrease compared with that on admission (Table 2). This decrease may be due to the fact that albumin degradation is more than synthesis during the clinical course of the disease. At the time of discharge, chest CT scans showed that the acute exudative lesions were completely absorbed in only about 20% of the patients (Table 1).

During follow-up after discharge, 5.6% of the 72 moderate survivors had the re-positive NAT results, and the remaining survivors had the negative NAT results. Chest CT images were negative in more than 70% of the 68 survivors and were further improved in the remaining survivors 4 weeks after discharge (Table 1). This suggests that the complete absorption of acute exudative lesions on chest CT scan need some time.

The 4 patients with re-positive NAT results were asked to return to the hospital for treatment. They had no symptoms, no abnormal laboratory results, and no exacerbated chest CT findings compared with those at discharge. After undergoing the median length of re-hospital stay of 8.5 days, and they were re-discharged. During subsequent follow-up, the 4 patients had no recurrence. These findings indicate that at least a small proportion of recovered patients still may be virus carriers and require an additional round of viral detection and quarantine. It has been reported that a proportion of COVID-19 patients experience a recurrence of positive NAT results after discharging from the hospital [22–25]. In our study, the negative NAT results might be false-negative at discharge, which may be associated with the following possibilities. Firstly, we cannot rule out the possibility that inadequate oropharyngeal swab specimens due to poor sampling techniques led to false-negative results [26–28]. Secondly, during treatment, the virus may be significantly controlled or removed from the upper respiratory tract, making it difficult to collect from the throat; however, this does not mean that the virus in the lower respiratory tract has also been completely removed. After stopping treatment for some time, the virus appeared again in the upper respiratory tract and was detected positive [22, 25]. Finally, the sensitivity of nucleic acid testing or test reliability may be also related to false-negative result; therefore, an additional RT-PCR test should be considered using a kit from a different manufacturer among discharged patients in the future [23, 26, 27].

Our study has several limitations. First, the total sample size is small. Second, the clinical process and management of severe/critical patients were not analyzed due to only 6 severe/critical cases on admission. Third, the NAT test for SARS-CoV-2 and laboratory tests were not performed 28 days after discharge so that no more follow-up data can be collected.

In conclusion, the data presented in this study show that the moderate patients with COVID-19 have a good prognosis by the active treatment aimed at symptom improvement. Moreover, a small proportion of the recovered moderate patients still may be virus carriers and require an additional round of viral detection.

## Supporting information

S1 Fig Chest CT images. The representative chest CT images of the patients with re-positive NAT results. A 62-year-old man showed bilateral ground-glass opacity and subsegmental areas of consolidation on admission (A), the above lesion improved significantly at discharge (B), the lesion that was not improved compared with Figure B on re-admission (C), and the lesion that was improved compared with Figure C at re-discharge (D), and the lesion absorbed completely 4 weeks after re-discharge (E). NAT results, nucleic acid test results for SARS-CoV-2.
(TIF)

S1 Table Demographics and baseline characteristics of 79 patients with COVID-19. (DOCX)

S2 Table Clinical manifestations of 79 patients with COVID-19. (DOCX)

S3 Table Treatment regimen of 79 patients with COVID-19. (DOCX)

S4 Table Demographics and baseline characteristics of 72 moderate survivors with COVID-19. (DOCX)

S5 Table Laboratory characteristics of 4 patients with re-positive NAT results. (DOCX)
