# Supplementary figures and images for: Clinical course and management of 73 hospitalized moderate patients with COVID-19 outside Wuhan
Source: PLoS One. 2021 May 13;16(5):e0249655. doi: 10.1371/journal.pone.0249655 (PMC8118515; doi:10.1371/journal.pone.0249655)

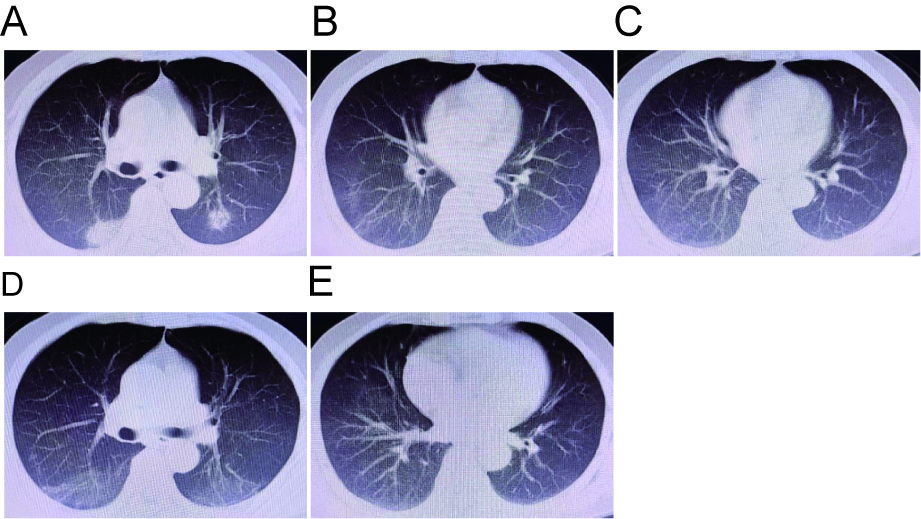

Supplement: S1 Fig — The representative chest CT images of the patients with re-positive NAT results. A 62-year-old man showed bilateral ground-glass opacity and subsegmental areas of consolidation on admission (A), the above lesion improved significantly at discharge (B), the lesion that was not improved compared with Figure B on re-admission (C), and the lesion that was improved compared with Figure C at re-discharge (D), and the lesion absorbed completely 4 weeks after re-discharge (E). NAT results, nucleic acid test results for SARS-CoV-2. (TIF) [file pone.0249655.s001.tif]
