# Supplementary material for: Clinical course and management of 73 hospitalized moderate patients with COVID-19 outside Wuhan
Source: PLoS One. 2021 May 13;16(5):e0249655. doi: 10.1371/journal.pone.0249655 (PMC8118515; doi:10.1371/journal.pone.0249655)
Supplement: S1 Table — (DOCX) [file pone.0249655.s002.docx]

S1 Table. Demographics and baseline characteristics of 79 patients with COVID-19

| Items | All patients  （n=79） | Moderate  （n=73） | Severe/ Critical  （n=5/1） |
| --- | --- | --- | --- |
| Age-yrs | 48.0 (39.0-59.0) | 47.0 (38.5-57.5) | 57.5 (37.5-63.0) |
| Sex-n (%) |  |  |  |
| Female | 37 (46.8) | 35 (47.9) | 1/1 (33.3) |
| Male | 42 (53.2) | 38 (52.1) | 4/0 (66.7) |
| Exposure history-n (%) |  |  |  |
| Familiar/cluster infections | 34 (43.0) | 33 (45.2) | 0/1 (16.7) |
| Community infections | 45 (57.0) | 40 (54.8) | 5/0 (83.3) |
| Occupation-n (%) |  |  |  |
| Agricultural worker | 33 (41.8) | 30 (41.1) | 2/1 (50.0) |
| Employee | 46 (58.2) | 43 (58.9) | 3/0 (50.0) |
| Smokers-n (%) | 10 (12.7) | 9 (12.3) | 1/0 (16.7) |
| Comorbidity-n (%) | 19 (24.1) | 17 (23.3) | 1/1 (33.3) |
| Hypertension | 13 (16.5) | 11 (15.1) | 1/1 (33.3) |
| Diabetes | 4 (5.1) | 4 (5.5) | 0/0 (0.0) |
| Chronic obstructive pulmonary disease | 2 (2.5) | 2 (2.7) | 0/0 (0.0) |
| Chronic renal disease | 2 (2.5) | 2 (2.7) | 0/0 (0.0) |
| Chronic heart disease | 1 (1.3) | 1 (1.4) | 0/0 (0.0) |
| Hypothyroidism | 1 (1.3) | 1 (1.4) | 0/0 (0.0) |
| Time from disease onset to admission-days | 5.0 (4.0-7.0) | 5.0 (4.0-7.5) | 5.5 (3.8-7.0) |
| Time from illness onset to the first positive result of NAT | 8.0 (6.0-11.0) | 8.0 (6.0-11.5) | 8.5 (7.5-9.5) |
| Survivors-n (%) | 74 (93.7) | 72 (98.6) | 2/0 (33.3) ** |
| Deceased-n (%) | 5 (6.3) | 1 (1.4) | 3/1 (66.7) ** |

Data are shown as median (IQR) or n (%).**P<0.01 vs the moderate patients. P values are from χ² test or Fisher’s exact test. COVID-19, coronavirus disease 2019; NAT, nucleic acid test for SARS-CoV-2; IQR, interquartile range.
