# Supplementary material for: Clinical course and management of 73 hospitalized moderate patients with COVID-19 outside Wuhan
Source: PLoS One. 2021 May 13;16(5):e0249655. doi: 10.1371/journal.pone.0249655 (PMC8118515; doi:10.1371/journal.pone.0249655)
Supplement: S2 Table — (DOCX) [file pone.0249655.s003.docx]

S2 Table. Clinical manifestations of 79 patients with COVID-19

| Items | All patients （n=79） | Moderate  （n=73） | Severe/ Critical  （n=5/1） |
| --- | --- | --- | --- |
| Cough-n (%) | 59 (74.7) | 53 (72.6) | 5/1 (100) |
| Fever-n (%) | 34 (43.0) | 28 (38.4) | 5/1 (100) ** |
| Chest tightness-n (%) | 31 (39.2) | 25 (34.2) | 5/1 (100) ** |
| Fatigue-n (%) | 20 (25.3) | 16 (21.9) | 3/1 (66.7) * |
| Dizzy/Headache -n (%) | 9 (11.4) | 9 (12.3) | 0/0 (0.0) |
| Nasal obstruction-n (%) | 8 (10.1) | 6 (8.2) | 1/1 (33.3) |
| Chill-n (%) | 2 (2.5) | 2 (2.7) | 0/0 (0.0) |
| Runny nose-n (%)t | 1 (1.3) | 1 (1.4) | 0/0 (0.0) |
| Gastrointestinal symptoms-n (%) | 17 (21.5) | 14 (19.2) | 3/0 (50.0) |
| Anorexia | 8 (10.1) | 7 (9.6) | 1/0 (16.7) |
| Vomiting | 5 (6.3) | 5 (6.8) | 0/0 (0.0) |
| Diarrhea | 6 (7.6) | 4 (5.5) | 2/0 (33.3) |
| Nausea | 2 (2.5) | 2 (2.7) | 0/0 |
| Abdominal pain | 1 (1.3) | 1 (1.4) | 0/0 (13) |
| Complications-n (%) | 7 (8.9) | 1 (1.4) | 5/1 (100) ** |
| Acute respiratory distress syndrome | 6 (7.6) | 1 (1.4) | 4/1 (83.3) ** |
| Acute heart failure | 4 (5.1) | 1 (1.4) | 2/1 (50.0) ** |
| Secondary infection | 3 (3.8) | 0 (0.0) | 3/0 (50.0) ** |
| Shock | 3 (3.8) | 0 (0.0) | 2/1 (50.0) ** |
| Sepsis | 1 (1.3) | 0 (0.0) | 1/0 (16.7) |
| Chest CT findings-n (%) |  |  |  |
| Bilateral viral pneumonia | 59 (74.7) | 53 (72.6) | 5/1 (100) |
| Unilateral viral pneumonia | 20 (25.3) | 20 (27.4) | 0/0 (0.0) |
| Right lung | 12 | 12 | 0/0 (0.0) |
| Left lung | 8 | 8 | 0/0 (0.0) |

Data are shown as n (%).*P<0.05, **P<0.01 vs the moderate patients. P values are from χ² test or Fisher’s exact test. COVID-19, coronavirus disease 2019.
