# Supplementary material for: Clinical course and management of 73 hospitalized moderate patients with COVID-19 outside Wuhan
Source: PLoS One. 2021 May 13;16(5):e0249655. doi: 10.1371/journal.pone.0249655 (PMC8118515; doi:10.1371/journal.pone.0249655)
Supplement: S3 Table — (DOCX) [file pone.0249655.s004.docx]

S3 Table. Treatment regimen of 79 patients with COVID-19

| Items | All patients  （n=79） | Moderate  （n=73） | Severe/ Critical  （n=5/1） | P value |
| --- | --- | --- | --- | --- |
| Antiviral treatment-n (%) | 79 (100.0) | 73 (100.0) | 5/1 (100.0) | - |
| Corticosteroid treatment-n (%) | 32 (40.5) | 26 (35.6) | 5/1 (100.0) | 0.003 |
| Antibacterial treatment-n (%) | 39 (49.4) | 33 (45.2) | 5/1 (100.0) | 0.012 |
| Oxygen support-n (%) |  |  |  |  |
| Nasal cannula | 41(51.9) | 41(56.2) | 0 (0.0) | 0.010 |
| Mechanical ventilation | 7 (8.9) | 1 (1.4) | 5/1 (100.0) | 0.000 |
| Traditional chinese medicine-n (%) | 73 (92.4) | 67 (91.8) | 5/1 (100.0) | 1.000 |

Data are shown as n (%). P values comparing moderate and severe/critical patients are from χ² test, or Fisher’s exact tes. COVID-19, coronavirus disease 2019. Antiviral treatment: combination of interferon-α and lopinavir/ ritonavir tablets or combination of interferon-α and abidol or combination of interferon-α and ribavirin. Corticosteroid treatment: methylprednisolone or budesonide. Antibacterial treatment: cephalosporins or quinolones. Traditional Chinese medicine: Lianhua Qingwen granules or Qingfei Paidu decoction or Cold Dampness and Stagnant Lung decoction.
