# Supplementary material for: Clinical course and management of 73 hospitalized moderate patients with COVID-19 outside Wuhan
Source: PLoS One. 2021 May 13;16(5):e0249655. doi: 10.1371/journal.pone.0249655 (PMC8118515; doi:10.1371/journal.pone.0249655)
Supplement: S4 Table — (DOCX) [file pone.0249655.s005.docx]

S4 Table. Demographics and baseline characteristics of 72 moderate survivors with COVID-19

| Items | Negative NAT (n=68) after discharge | Re-positive NAT (n=4) after discharge | P-value |
| --- | --- | --- | --- |
| Age-yrs | 46.0 (37.3-55.8) | 60.0 (41.3-72.8) | 0.201 |
| Sex-n (%) |  |  |  |
| Female | 31 (45.6) | 3 (75.0) | 0.338 |
| Male | 37 (54.4) | 1 (25.0) | 0.338 |
| Exposure history-n (%) |  |  |  |
| Familiar/cluster infections | 30 (41.1) | 2 (50.0) | 1.000 |
| Community infections | 38 (55.9) | 2 (50.0) | 1.000 |
| Occupation-n (%) |  |  |  |
| Agricultural worker | 25 (36.8) | 4 (100.0) | 0.023 |
| Employee | 43 (63.2) | 0 (0.0) | 0.023 |
| Smokers-n (%) | 9 (12.3) | 0 (0.0) | 1.000 |
| Comorbidity-n (%) | 16 (23.5) | 1 (25.0) | 1.000 |
| Hypertension | 9 (13.2) | 1 (25.0) | 0.458 |
| Diabetes | 3 (4.4) | 1 (25.0) | 0.208 |
| Chronic obstructive pulmonary disease | 2 (2.9) | 0 (0.0) | 1.000 |
| Chronic renal disease | 2 (2.9) | 0 (0.0) | 1.000 |
| Chronic heart disease | 1 (1.5) | 0 (0.0) | 1.000 |
| Hypothyroidism | 1 (1.4) | 0 (0.0) | 1.000 |
| Time from disease onset to admission-days | 5.0 (4.0-7.8) | 6.5 (3.8-7.8) | 0.692 |
| Time from illness onset to the first positive result of NAT | 8.0 (6.0-11.0) | 8.5 (7.3-11.3) | 0.504 |
| Survivors-n (%) | 68 (100.0) | 4 (100.0) | **-** |
| Deceased-n (%) | 0 (0.0) | 0 (0.0) | **-** |
| Re-hospitalization time-days | - | 8.5 (6.5-10.5) | - |

Data are shown as median (IQR) or n (%). NAT, nucleic acid test for SARS-CoV-2; IQR, interquartile range.
