# Supplementary material for: Clinical course and management of 73 hospitalized moderate patients with COVID-19 outside Wuhan
Source: PLoS One. 2021 May 13;16(5):e0249655. doi: 10.1371/journal.pone.0249655 (PMC8118515; doi:10.1371/journal.pone.0249655)
Supplement: S5 Table — (DOCX) [file pone.0249655.s006.docx]

S5 Table. Laboratory characteristics of 4 patients with re-positive NAT results

| Items | Patients on admission  （n=4） | Patients on discharge  （n=4） | Patients on re-admission Patients on re-discharge  （n=4） （n=4） | P-value |
| --- | --- | --- | --- | --- |
| **Blood routine** |  |  |  |  |
| Leucocytes (×10^9^/L; normal range 3.5-9.8) | 4.1 (3.8-5.3) | 5.3 (4.9-6.2) | 5.3 (4.6-6.7) 5.5 (4.4-7.1) | 1.000 |
| Neutrophils (×109/L; normal range 1.8-6.3) | 2.8 (2.3-3.9) | 3.2 (2.8-3.8) | 3.5 (3.3-4.8) 3.9 (2.6-5.9) | 0. 248 |
| Neutrophil percentage-(%) (normal range 40-75) | 69.5 (59.3-73.9) | 61.5 (51.9-64.7.3) | 67.4 (60.9-72.3) 64.9 (54.7-72.0) | 0.149 |
| Lymphocytes (×10^9^/L; normal range 1.1-3.2) | 1.0 (0.7-1.2) | 1.6 (1.2-2.1) | 1.5 (1.1-1.8) 1.5 (1.4-1.6) | 0.564 |
| Lymphocyte percentage-(%) (normal range 20-50) | 21.3 (17.0-29.9) | 27.2 (25.8-38.0) | 21.8 (20.8-25.6) 25.2 (17.8-35.5) | 0.083 |
| Platelets (×10^9^/L; normal range 125-350) | 269.0 (256.8-312.8) | 229.0 (178.3-278.3) | 247.0 (170.0-263.3) 238.5 (166.3-276.3) | 0.773 |
| Haemoglobin (normal range 115-150 g/L) | 133.3 (99.0-139.3) | 131.5 (122.5-139.0) | 142.5 (138.3-148.3) 132.0 (114.0-150.8) | 0.083 |
| **Infection biomarkers** |  |  |  |  |
| C-reactive protein (mg/L; normal range 0.0-8.0) | 29.5 (5.3-46.6) | 0.9 (0.4-1.1) | 1.2 (0.5-5.4) 1.3 (0.9-3.4) | 0.564 |
| Procalcitonin (ng/mL; normal range 0.0-0.5) | 0.12 (0.05-0.19) | 0.05 (0.05-0.09) | 0.07 (0.05-0.13) 0.07 (0.05-0.15) | 0.457 |
| **Coagulation function** |  |  |  |  |
| Prothrombin time (s; normal range 9.0-15.0) | 11.0 (10.3-12.3) | 11.9 (11.6-12.3) | 10.7 (9.7-11.7) 11.03(11.0-12.5) | 0.080 |
| Activated partial thromboplastin time (s; normal range 22.0-45.0) | 28.4 (26.4-31.1) | 26.1 (24.1-27.9) | 26.0 (25.8-27.7) 26.0 (23.6-28.7) | 0.773 |
| D-dimer (µg/mL; normal range 0.0-0.5) | 0.08 (0.03-0.70) | 0.41 (0.07-1.11) | 0.07 (0.03-0.77) 0.14 (0.09-0.64) | 0.309 |
| **Blood biochemistry** |  |  |  |  |
| Fasting blood glucose (mmol/L; normal range 3.9-6.1) | 5.4 (4.9-6.0) | 4.24 (4.0-5.9) | 5.4 (4.4-6.2) 4.8 (4.5-5.6) | 0.386 |
| Albumin (g/L; normal range 35.0-52.0) | 38.1(34.4-40.2) | 35.4 (30.2-42.3) | 38.5 (32.1-41.8) 36.2 (30.7-39.4) | 0.564 |
| Alanine aminotransferase (U/L; normal range 9-50) | 18.7 (6.7-26.8) | 17.6 (6.0-31.4) | 14.9 (6.7-22.3) 19.1 (11.3-28.3) | 0.883 |
| Aspartate aminotransferase (U/L; normal range 15-40) | 26.4 (16.1-37.2) | 18.7 (14.0-20.3) | 16.5 (15.2-19.1) 20.2 (15.0-24.6) | 0.564 |
| GGT (U/L; normal range 11-61) | 16.6 (15.8-28.6) | 20.9 (13.7-26.7） | 20.5 (16.4-26.3) 26.2 (14.5-34.6) | 0.773 |
| Total bilirubin (μmol/L; normal range 5.1-23.0) | 8.0 (7.0-15.1) | 9.4 (6.3-14.1) | 8.9 (7.5-9.7) 6.5 (4.5-8.9) | 0.773 |
| Creatine kinase (U/L; normal range 26-174) | 51.7 (44.0-58.6) | 34.3 (11.7-78.4) | 53.6 (27.5-82.5) 44.8 (28.1-87.9) | 0.386 |
| Creatine kinase-MB(U/L; normal range 3-25) | 9.3 (6.2-14.6) | 10.0 (5.0-15.7) | 12.9 (8.6-15.8) 7.3 (6.9-12.1) | 0.564 |
| Lactate dehydrogenase (U/L; normal range 109-245) | 223.9 (148.6-399.9) | 173.2 (169.3-242.8) | 187.1.6 (159.9-244.5) 195.5 (166.6-222.5) | 0.773 |
| Serum creatinine (μmol/L; normal range 32-106) | 62.9 (55.5-74.9) | 55.8 (54.8-58.4) | 55.9 (54.4-57.6) 59.9 (49.8-76.9) | 0.773 |
| Blood urea nitrogen (mmol/L; normal range | 3.6 (3.1-4.3) | 5.4 (3.8-6.6) | 4.7 (3.4-5.7) 3.5 (2.9-4.9) | 0.564 |
| 1.5-7.5) |  |  |  |  |

Data are shown as median (IQR). P values comparing patients on discharge and patients on re-admission are from Mann-Whitney U test. NAT, nucleic acid test for SARS-CoV-2; IQR, interquartile range.
